# Supplementary material for: Continuous signaling of CD79b and CD19 is required for the fitness of Burkitt lymphoma B cells
Source: EMBO J. 2018 Apr 18;37(11):e97980. doi: 10.15252/embj.201797980 (PMC5983214; doi:10.15252/embj.201797980)
Supplement: Supplementary file 1 — Appendix [file EMBJ-37-e97980-s001.pdf]

Table of contents

Appendix Figure S1

Appendix Figure S2

Appendix Figure S3

Appendix Figure S4

Appendix Figure S5

Appendix Figure S6

Appendix Table S1

Appendix Table S2

Appendix Figure legends

Appendix Figure S1.

(**A and C**) A schematic diagram showing the route for the generation of single- and multi-BCR component KOs from WT Ramos B cells by the CRISPR/Cas9 method. **(B and D)** Expression of the indicated BCR component in WT and the different Ramos KO cells as measured by flow cytometry after staining with the corresponding antibodies. **(E)** Western blot analysis of the expression of the indicated BCR component in WT and the different KO Ramos cells. One clone is used for each genotype.

Appendix Figure S2.

Cell proliferation assay of WT and BCR component KO Ramos cells using CytoTell™ Ultragreen. The data are representative of three independent experiments. One clone is used for each genotype.

Appendix Figure S3.

Growth competition between BCR component KO cells and gWT Ramos cells. The GFP^+ viable cells were single-cells sorted into 96-well plates 48h post transfection of the CRISPR/Cas9 KO plasmid. The sorted cells were cultured for 10 days and then transferred into larger wells for expansion for another 4 days. After the deletion of the target gene has been confirmed by staining with the corresponding antibodies, the freshly generated single colonies were immediately used for growth competition assay. The competition between GFP^- WT and gWT cells serves as a control. The data represent the mean and standard error of a minimum of three independent experiments. Differences between the WT and KO cells were evaluated by first performing the multiple t-test for each day without assuming the same SD and the multiple t-test results were then further analyzed using the False Discovery Rate (FDR, Q) approach with Two-stage linear step-up procedure. Data showed significant difference ($Q<0.01$) were marked by stars. One clone (Different than those used in Fig 1) is used for each genotype.

Appendix Figure S4.

(A-D) Calcium responses of WT and the indicated BCR component KO Ramos cells upon the stimulation with anti-Igα antibodies. HL KO = HC/LC double KO; HLα KO = HC/LC , Igα triple KO; HLβ KO = HC/LC, Igβ triple KO. One clone is used for each genotype.

Appendix Figure S5.

(A) A schematic diagram showing the generation of SLP65 KO from HL KO Ramos B cells by the CRISPR/Cas9 method. **(B)** Western blot analysis of SLP65 expression in HL double KO and HLSLP65 triple KO Ramos cells. The staining for GAPDH served as loading control. One clone is used for each genotype.

Appendix Figure S6.

(A) A schematic diagram showing the generation of CD19 or CD81 deficient cells from HL KO Ramos B cells by the CRISPR/Cas9 method. **(B)** FACScan analysis of CD19 or CD81 expression on HL KO, HLCD19 KO or HLCD81 KO Ramos B cells as measured by flow cytometry. One clone is used for HLKO. HLCD19KO and HLCD81 KO are batch sorted.

Fig. S1

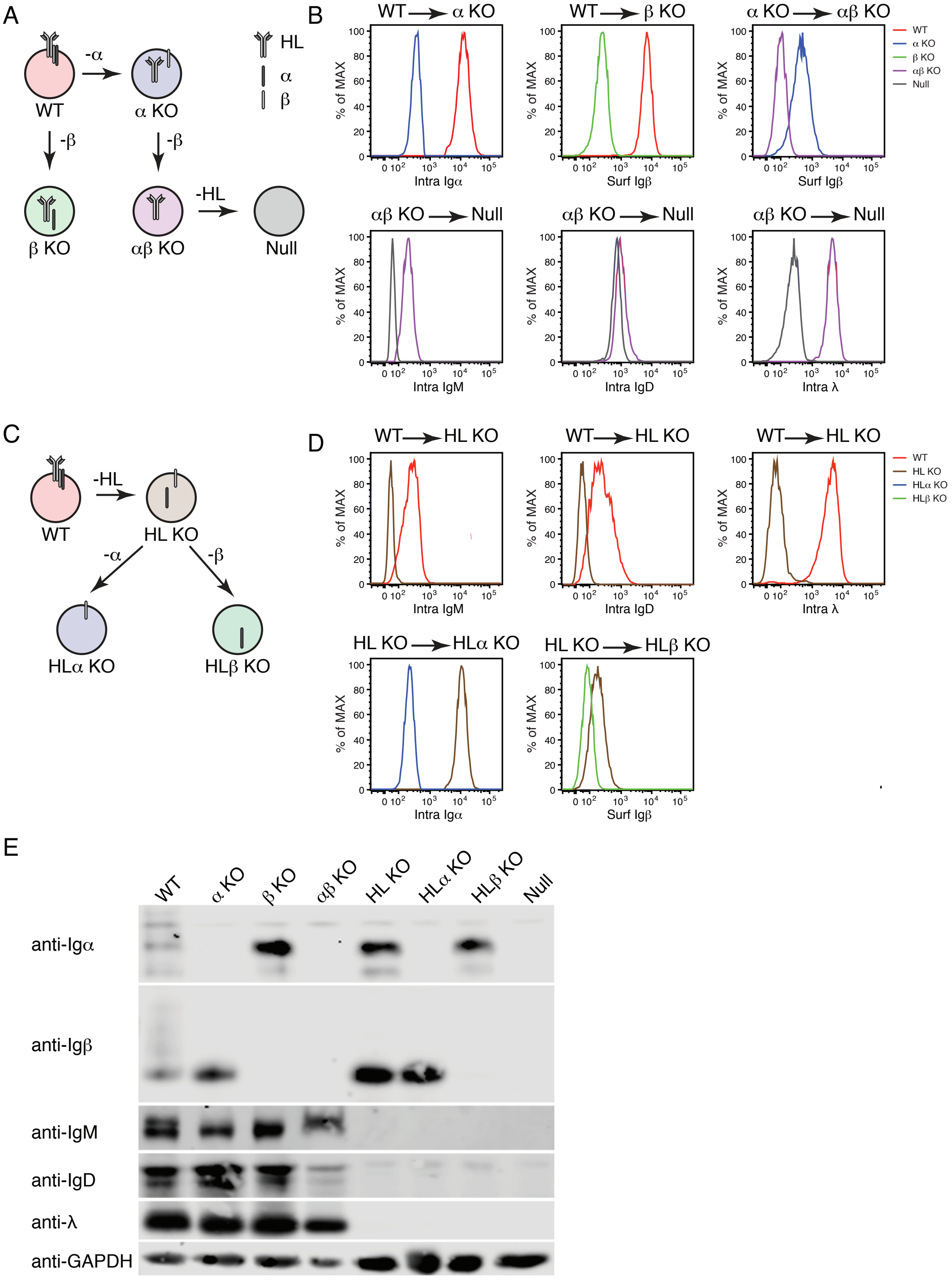
A
WT
α KO
β KO
αβ KO
Null
HL
α
β
-α
-β
-HL
B
WT → α KO
WT → β KO
α KO → αβ KO
αβ KO → Null
% of MAX
Intra Igα
Surf Igβ
Intra IgM
Intra IgD
Intra λ
WT
α KO
β KO
αβ KO
Null
C
HL KO
HLα KO
HLβ KO
D
WT → HL KO
HL KO → HLα KO
HL KO → HLβ KO
E
anti-Igα
anti-Igβ
anti-IgM
anti-IgD
anti-λ
anti-GAPDH

Fig. S2

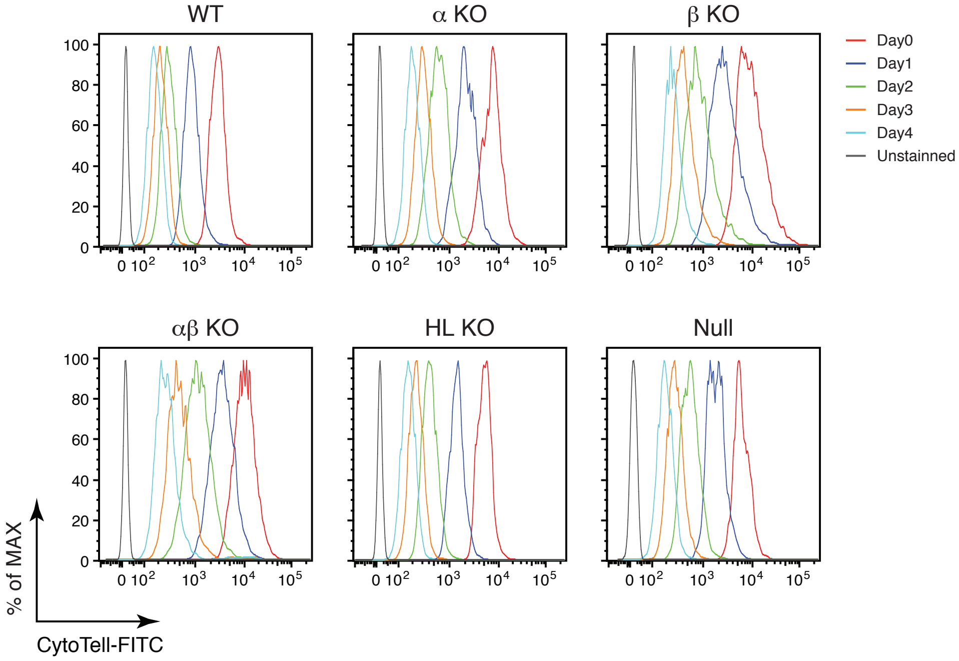
WT
α KO
β KO
αβ KO
HL KO
Null
Day0
Day1
Day2
Day3
Day4
Unstainned
% of MAX
CytoTell-FITC
0 20 40 60 80 100
0 10^2 10^3 10^4 10^5

Fig. S3

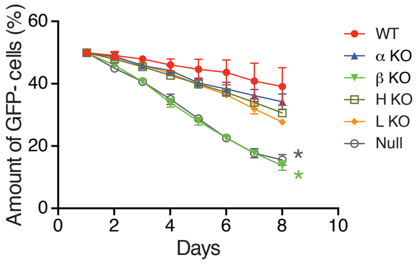
Amount of GFP- cells (%)
60
40
20
0
0
2
4
6
8
10
Days
WT
α KO
β KO
H KO
L KO
Null
*
*

Fig. S4

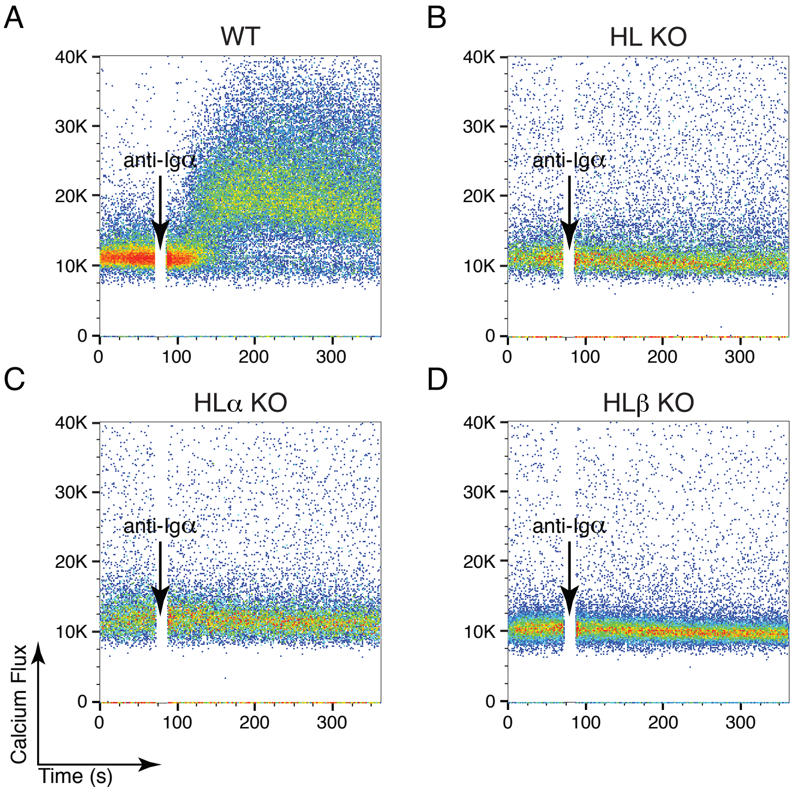
A
WT
40K
30K
20K
10K
0
anti-Igα
0
100
200
300
B
HL KO
40K
30K
20K
10K
0
anti-Igα
0
100
200
300
C
HLα KO
40K
30K
20K
10K
0
anti-Igα
0
100
200
300
D
HLβ KO
40K
30K
20K
10K
0
anti-Igα
0
100
200
300
Calcium Flux
Time (s)

Fig. S5

A

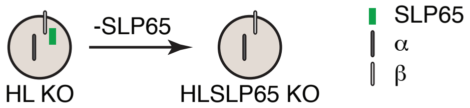

-SLP65
HL KO
HLSLP65 KO
SLP65
α
β

B

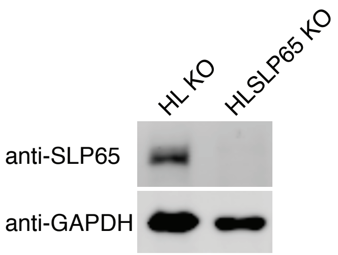

HL KO
HLSLP65 KO
anti-SLP65
anti-GAPDH

Fig. S6

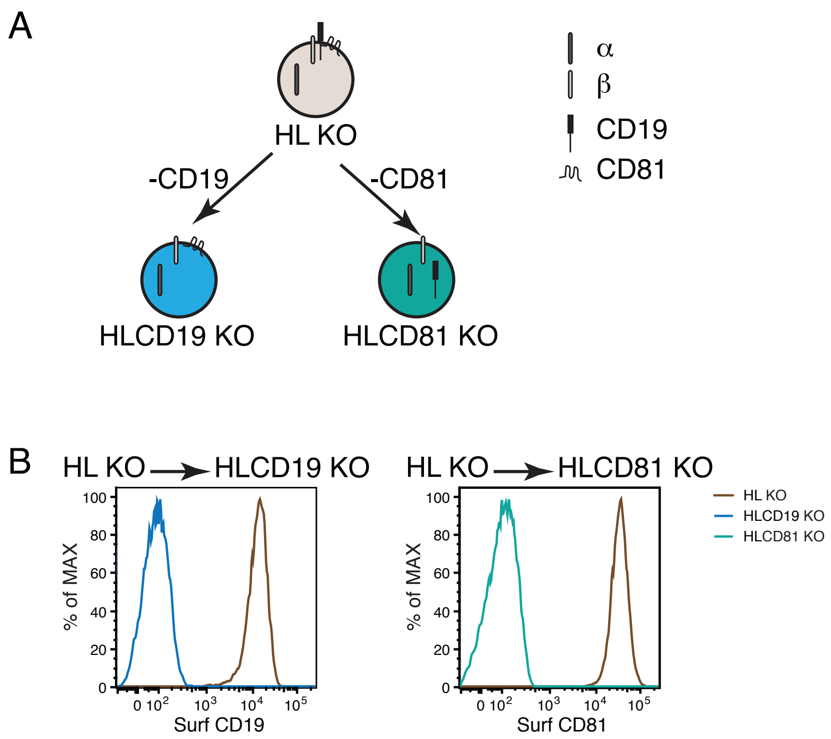
A
HL KO
-CD19
-CD81
HLCD19 KO
HLCD81 KO
α
β
CD19
CD81
B
HL KO → HLCD19 KO
HL KO → HLCD81 KO
% of MAX
100
80
60
40
20
0
0 10^2 10^3 10^4 10^5
Surf CD19
Surf CD81
HL KO
HLCD19 KO
HLCD81 KO

Appendix Table S1. Information of CRISPR/Cas9 KO plasmids

Target gene	Description
Igα	Purchased from Santa Cruz
Igβ	Purchased from Santa Cruz
mIg Heavy chain	sgRNA 5´-CAGCTACAGCAGTGGGGCGC-3´
mIg Light chain	sgRNA 5´-GGGGTTTCTAATCGCTTCTC-3´
SLP65	Purchased from Santa Cruz
CD19	sgRNA 5´-AAGCGGGGACTCCCGAGACC-3´
CD81	Purchased from Santa Cruz

Appendix Table S2. Information of primers for cloning

Name	Sequences 5´-3´	Template	Final plasmid description
Igβ65F	GTTTGGGGTCGGGGACAG	Ramos cDNA	pJET-Igβ
Igβ704 R	CCCGTCCATCCCCAGAGA		
pMIGIgβ_F	ATTAGATCTCTCGAGCCACATGGCCAGGCTGGCGTTG	pJET-Igβ	PCR fragments were fused into the Xho I linearized pMIG to obtain pMIG-Igβ
pMIGIgβ_R	GCGGAATTCGTTAACTCACTCCTGGCCTGGGTG		
pMIGHAIgβ_F1	TATCCATATGATGTTCCAGATTATGCTGCCAGATCGGAGGACCGG	pMIG-Igβ	HA tagged Igβ plasmid: pMIG-HA-Igβ
pMIG_R1	CCGTAGAAAAGATCAAAG		
pMIG_F2	TGATCTTTTCTACGGGGT	pMIG-Igβ	
pMIGHAIgβ _R2	AACATCATATGGATATGCTGGTACTGGCTCAGC		
Igα74F	GCCACTGGAGCCCATCTC	Ramos cDNA	pJET-Igα
Igα771R	CCTGGCAGGAGTAGGGGT		
pMIGIgα_F	ATTAGATCTCTCGAGCCACATGCCTGGGGGTCCAGGA	pJET-Igα	PCR fragments were fused into the Xho I linearized pMIG to obtain pMIG-Igα
pMIGIgα_R	GCGGAATTCGTTAACTCACGGCTTCTCCAGCTG		
pMIGIgβAt_F1	CCTATCTTCCTGCTGAGGAAACGATGGCAGAAC	pMIG-Igα	Two PCR fragments were fused together to

pMIG_R1	See above		obtain pMIG-HA-Igβ- Igα tail
pMIG_F2	See above	pMIG-HA-Igβ	
pMIGIgβAt_R2	CTGCCATCGTTTCCTCAGCAGGAAGATAGGCAC		
IgβY196F_F	GGAAGATCACACCTTCGAGGGCCTGGACA	pMIG-HA-Igβ	Igβ ITAM Y196 was mutated into F
IgβY196F_R	TGTCCAGGCCCTCGAAGGTGTGATCTTCC		
IgβY207F_F	CCAGACAGCCACCTTTGAGGACATAGTGA	pMIG-HA-Igβ	Igβ ITAM Y207 was mutated into F
IgβY207F_R	TCACTATGTCCTCAAAGGTGGCTGTCTGG		
IgβTailL_F1	AAGGATGACAGCAAGTGAGTTAACGAATTCCGC	pMIG-HA-Igβ	Two PCR fragments were fused together to obtain pMIG-HA-Igβ tail less
pMIG_R1	See above		
pMIG_F2	See above	pMIG-HA-Igβ	
IgβTailL_R2	GAATTCGTTAACTCACTTGCTGTCATCCTTGTC		
CD19_F1	CCCCGGAGAGTCTGACCA	Ramos cDNA	pJET- CD19
CD19_R1	AGGAGATCCAGGCTGGCC		
pMIGCD19_F	ATTAGATCTCTCGAGCCACATGCCACCTCCTCGCCTC	pJET- CD19	PCR fragments were fused into the Xho I linearized pMIG to obtain pMIG-CD19
pMIGCD19_R	GCGGAATTCGTTAACTCACCTGGTGCTCCAGGT		
