# Supplementary material for: Continuous signaling of CD79b and CD19 is required for the fitness of Burkitt lymphoma B cells
Source: EMBO J. 2018 Apr 18;37(11):e97980. doi: 10.15252/embj.201797980 (PMC5983214; doi:10.15252/embj.201797980)
Supplement: Supplementary file 2 — Expanded View Figures PDF [file EMBJ-37-e97980-s002.pdf]

Expanded View Figures

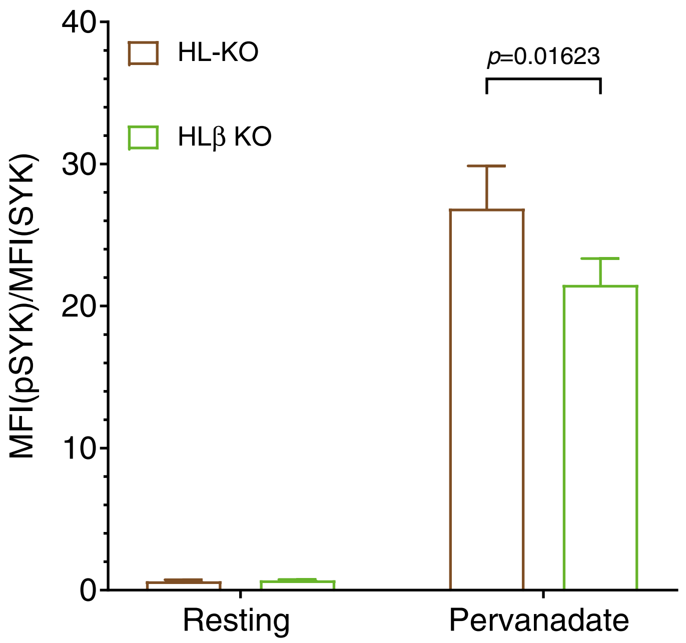

Figure EV1. Flow cytometry analysis of phosphoSyk.

Normalized intracellular phosphoSyk levels in resting and pervanadate treated HL double KO and HLβ triple KO Ramos cells. The data represent the mean and the standard deviation of three independent experiments. *P*-values were calculated by paired *t*-test. One clone is used for each genotype.

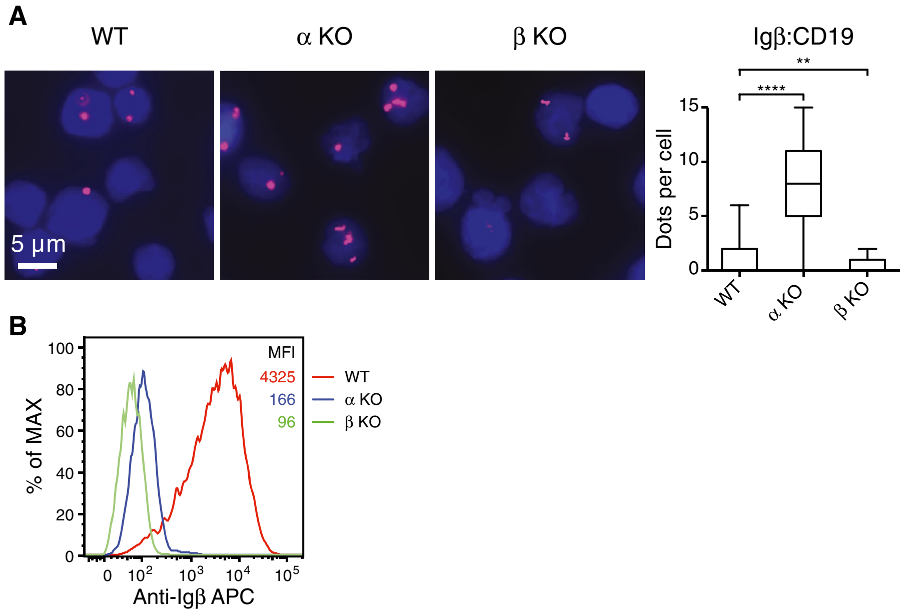

Figure EV2. Fab-PLA analysis of the Igβ:CD19 proximity on BCR-negative DG75 cells.

A Measurement of the Igβ:CD19 proximity on WT, Igα KO or Igβ KO DG75 B cells by Fab-PLA. The data are representative of three independent experiments. *P*-values were calculated by non-parametric Mann–Whitney test. ** indicates $P < 0.01$, **** indicates $P < 0.0001$. In the box plot, horizontal lines in the middle represents median, box limits represent 25% to 75%, and lower and upper whisker represent min. and max. data range respectively.

B Expression levels of Igβ in WT, HLα KO, and HLβ KO DG75 cells were determined by flow cytometry. One clone is used for each genotype.

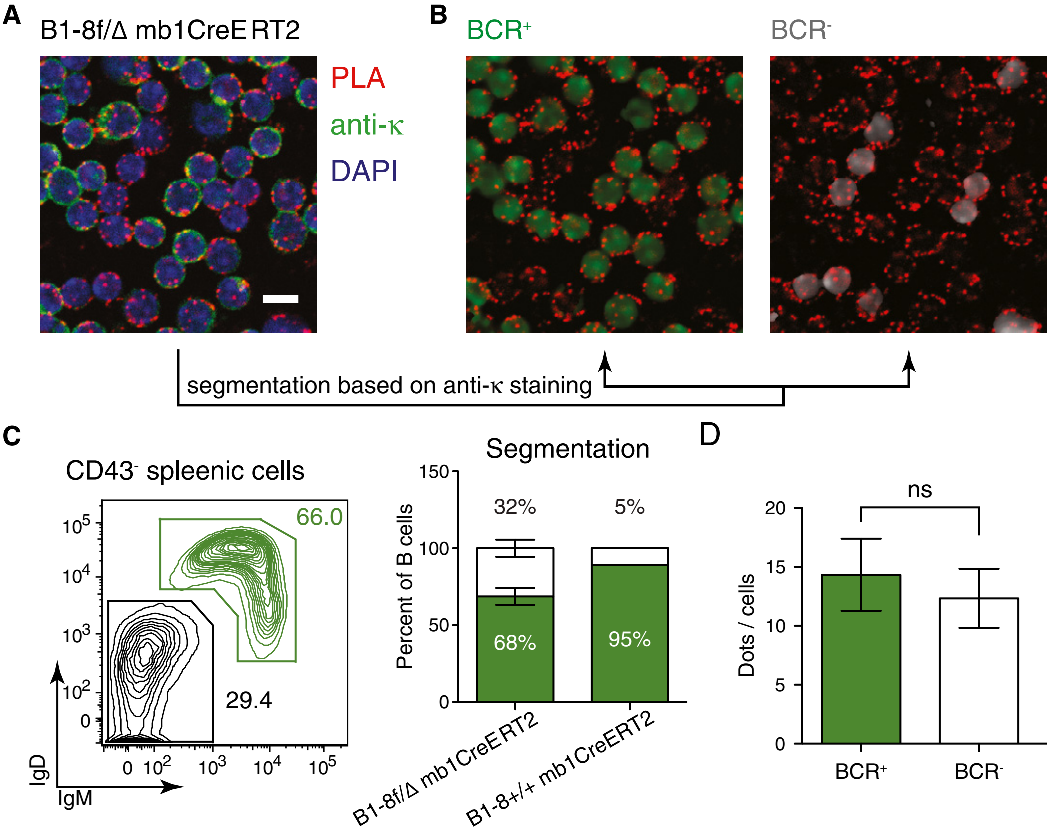

Figure EV3. 1-PLA analysis of the Igβ:CD19 proximity on splenic B cells from a tamoxifen treated B1-8f/Δ mb1CreERT2 mouse.

A Representative microscopic image showing splenic B cells from a tamoxifen treated B1-8f/Δ mb1CreERT2 mouse. The different colors indicate anti-kappa LC staining (green), DAPI staining (blue), and the PLA signals (red). Scale bar: 5 μm.

B For PLA signal quantification, the image in (A) was segmented *in silico* to a BCR^+ (green) and BCR^- (gray) cell population.

C Left panel shows a representative flow cytometry analysis to determine the percentage of BCR^+ cells (IgM^+IgD^+) and BCR^- cells (IgM^-IgD^-) of B cells from the same spleen. Right panel shows the percentage of BCR^+ and BCR^- cells after *in silico* segmentation. It also shows that the percentage of BCR^+ and BCR^- cells of splenic cells from B1-8f/Δ mb1CreERT2 mouse determined by *in silico* segmentation is as expected. Data represent the mean and the standard error of six experiments with each of them counting 50–200 cells.

D Quantification of the PLA signals in BCR^+ and BCR^- cells on the segmented images. Data represent the mean and the standard error of six experiments with each of them counting 50–200 cells. *P*-values were calculated by Wilcoxon signed rank test.

 © 2018 The Authors
